# Acupuncture for Lateral Epicondylitis: A Systematic Review

**DOI:** 10.1155/2015/861849

**Published:** 2015-12-30

**Authors:** Hongzhi Tang, Huaying Fan, Jiao Chen, Mingxiao Yang, Xuebing Yi, Guogang Dai, Junrong Chen, Liugang Tang, Haibo Rong, Junhua Wu, Fanrong Liang

**Affiliations:** ^1^Sichuan Orthopaedic Hospital, Chengdu, Sichuan 610041, China; ^2^Chengdu University of Traditional Chinese Medicine, Chengdu, Sichuan 610075, China

## Abstract

*Objective*. This systematic review aimed to assess the effectiveness and safety of acupuncture for lateral epicondylitis (LE).* Methods*. Seven databases and the WHO International Clinical Trials Registry Platform Search Portal were searched to identify relevant studies. The data were extracted and assessed by two independent authors, and Review Manager Software (V.5.3) was used for data synthesis with effect estimate presented as standard mean difference (SMD) and mean difference (MD) with a 95% confidence interval. The Grading of Recommendations Assessment, Development, and Evaluation (GRADE) was used to assess the level of evidence.* Results*. Four RCTs with 309 participants were included with poor methodological quality. Participants who received acupuncture and acupuncture plus moxibustion with material insulation were likely to have an improvement in elbow functional status and/or myodynamia. The overall quality rated by GRADE was from very low to low. Two studies reported that the needle pain would be the main reason for the dropout.* Conclusion*. For the small number of included studies with poor methodological quality, no firm conclusion can be drawn regarding the effect of acupuncture of elbow functional status and myodynamia for LE. This trial is registered with CRD42015016199.

## 1. Introduction

Lateral epicondylitis (LE), also known as tennis elbow, is upper limbs associated musculoskeletal disorder and can be responsible for loss of function of the affected limb and substantial pain, which can have a major impact on patient's social and professional life [1]. It is estimated that the prevalence of LE ranges from 1% to 3% in the overall population [2], mainly occurring in those aged 45–54 years [3]. Activities that involve excessive and repetitive use of the forearm extensor, such as typing, tennis, badminton, and manual work, may cause LE [4]. The principal cause of LE is the degeneration of the proximal wrist extensor tendons [5].

LE not only is a major problem that causes prolonged recovery of functional disability and long-time pain that impact patient's daily life, but also produces a heavy economic burden as lost workdays and, in some patients, inability to work may last for several weeks [6, 7]. While a number of treatment methods, such as nonsteroidal anti-inflammatory drugs (NSAIDs), corticosteroid injections, exercise and mobilization, extracorporeal shock wave therapy, orthoses, and surgery, are used for LE, the lines of evidence of the effectiveness and safety of these therapy methods still remain uncertain [8–13].

As acupuncture is a green, simple, inexpensive, and helpful treatment which has been widely practiced in China and increasingly used in some Western countries, such as the Unite States, it has been accepted for treating musculoskeletal disease, especially for the functional disability and pain symptoms [14]. In recent years, a number of clinical trials have been conducted to assess the effectiveness and safety of acupuncture therapy for LE.

There were five systematic reviews that have been published during the last few years for assessing the effectiveness and safety of acupuncture for LE by evaluating the pain changes, but none of them draw a definitive conclusion on whether acupuncture is effective for LE or not. The systematic review published in 2002 by Green et al. [15] on lateral epicondylitis did not draw a conclusion on whether the acupuncture is effective for LE or not because of the small number of included trials and problems with methodology of the included trials, and the systematic review published in 2008 by Buchbinder et al. [16] also did not draw a specific conclusion because they thought there is conflicting evidence about the value of acupuncture for LE. However, the systematic review published in 2004 by Trinh et al. [17] suggested that acupuncture is effective for short-term pain relief for LE pain, and another systematic review published in 2005 by Bisset et al. [18] suggested that acupuncture is effective over placebo as treatment for LE in short-term outcomes, and the systematic review published by Gadau et al. in 2014 [19] also showed that acupuncture may be effective in the relief of LE pain up to a period of six months. Besides, although the systematic reviews published by Buchbinder et al. [16] and Gadau et al. [19] have also assessed the functional improvement or arm strength of LE, the conclusion still remains unclear because there was only one RCT included by assessing the functional improvement in review published by Buchbinder et al. and the author did not pool-analyze the included studies in the review published by Gadau et al.

Therefore, we can see in these systematic reviews that acupuncture has some effect on treating LE pain, but what remains unclear is whether acupuncture is effective and safe in improving the functional disability and changing the myodynamia, which seems to be important to improving patient's quality of life, or not. Thus, we decided to conduct the latest systematic review by evaluating the elbow functional status and myodynamia changes of the included trials to assess the effectiveness and safety of acupuncture for LE.

## 2. Method and Analysis

### 2.1. Search Method

The following 7 databases were electronically searched from their inception to 2015: EMBASE, PubMed, the Cochrane Library, China National Knowledge Infrastructure (CNKI), Chinese Scientific Journal Database (VIP database), Wanfang Database, and Chinese Biomedical Literature Database (Sinomed). For the last four Chinese databases, we only included the researches published on core journals, such as Chinese Acupuncture and Moxibustion. The search terms consisted of four parts: acupuncture (acupuncture, electroacupuncture, warm acupuncture, needle acupuncture, and manual acupuncture), LE (lateral epicondylitis, tennis elbow, lateral epicondyle, external humeral epicondylitis, and lateral humeral epicondylitis), and randomized controlled trial. The detailed search strategies are presented in the Appendix.

Besides, we also searched the WHO International Clinical Trials Registry Platform Search Portal that contains Current Controlled Trials, ClinicalTrials.gov, and Chinese Clinical Trial Register for ongoing or recently completed studies by using simple search combining acupuncture and LE.

### 2.2. Inclusion Criteria

#### 2.2.1. Type of Studies

All randomized controlled trials (RCTs) involving acupuncture for treating LE were included. Completed or ongoing trials were included in this review, as well as trials using only the two parallel designs.

#### 2.2.2. Type of Participants

Adult participants (≥18 years old) presenting with LE were included regardless of sex, race, or educational and economic status.

#### 2.2.3. Types of Interventions

Interventions in the treatment group included acupuncture, electroacupuncture, warm acupuncture, needle acupuncture, and manual acupuncture. Controlled interventions with sham acupuncture, placebo control, no treatment/waiting list control, or active treatment (e.g., nonsteroidal anti-inflammatory drugs and/or local injection of corticosteroids) were included. RCTs evaluating acupuncture combined with another treatment compared with that other treatment alone will also be included.

#### 2.2.4. Types of Outcome Measures

Studies that reported at least one clinical outcome related to LE were included. Studies reporting only physiological or laboratory parameters were excluded. The primary outcome was the elbow functional status. The secondary outcome was the myodynamia and adverse events.

### 2.3. Exclusion Criteria

#### 2.3.1. Other Types of Studies

Nonrandomized controlled trials, randomized crossover trials, retrospective studies, case studies, and review studies were excluded.

#### 2.3.2. Other Participants

Participants with severe physical or mental disease were excluded.

#### 2.3.3. Other Types of Interventions

We did not include trials in which points were stimulated without needle insertion (such as via laser stimulation, acupressure, or transcutaneous electrical nerve stimulation). Besides, RCTs that compare different forms of acupuncture or herbal medicine were excluded.

### 2.4. Study Identification and Data Extraction

Two reviewers (Hongzhi Tang and Huaying Fan) independently assessed the eligibility of the searched studies. The full-text articles that met the inclusion criteria were obtained, and the relevant references were retrieved according to predefined eligibility criteria. The data has been extracted by two reviewers (Jiao Chen and Huaying Fan) independently using a specially designed extraction form developed according to the Cochrane Handbook. The following factors were included in the data extraction form: participants' characteristics, study methods, interventions, and outcomes. We resolved any disagreements of study identification and data extraction by discussion and adjudication with a third reviewer (Hongzhi Tang). If any data were insufficient or unclear, the first or corresponding author for the study concerned would be contacted via E-mail or telephone to provide additional information.

### 2.5. Risk of Bias of the Included Studies

Two reviewers (Hongzhi Tang and Jiao Chen) assessed the included studies for bias risk according to the Cochrane Collaboration Risk of Bias Tool based on the following six separate domains: random sequence generation (selection bias), allocation concealment (selection bias), blinding of participants and personnel (performance bias), blinding of outcome assessment (detection bias), incomplete outcome data (attrition bias), and selective reporting (reporting bias). The assessments were categorized into three levels of bias: low risk, high risk, or unclear risk.

### 2.6. Data Synthesis

Meta-analysis was done by using Review Manager Software (V.5.3) developed by the Cochrane Collaboration. Trials are combined according to the type of intervention, type of outcome measure, and control. For the continuous data, the mean difference (MD) with 95% confidence intervals was used with random-effects model. For different studies assessed the elbow functional status and myodynamia in a variety of ways, according to the Cochrane Handbook, the standardized mean difference (SMD) was used to standardize the results of the studies to a uniform scale before they were combined. Heterogeneity among the included studies was assessed using chi-square and *I* ^2^ test. *I* ^2^ values indicate the degree of statistical heterogeneity. When *P* > 0.1, it was considered that there was no statistical heterogeneity among studies; if *P* ≤ 0.1, it was inverse. *I* ^2^ values less than 50% were accepted as homogeneous. If substantial heterogeneity was detected, we explored the reason for heterogeneity.

### 2.7. Quality of Evidence

We used the Grading of Recommendations Assessment, Development, and Evaluation (GRADE), which is a method of grading the level of evidence and is developed by the GRADE Working Group [24, 25], to assess the quality of evidence, and the GRADEpro software (version 3.6 for Windows, Grade Working Group) was used.

## 3. Results

### 3.1. Study Identification

**Figure 1:**
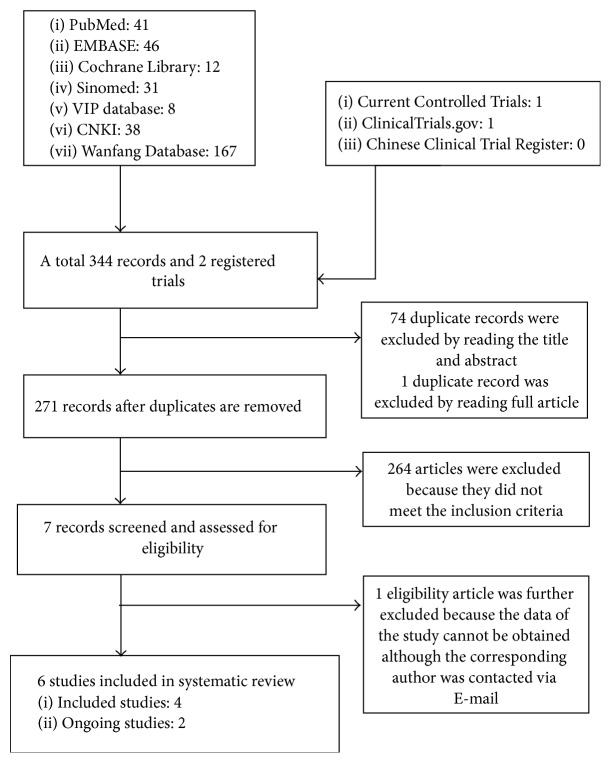
Flow diagram for search and selection of the included studies.

The flow of the literature search and selection process is shown in Figure 1. A total of 344 records and two registered trials were identified from the included databases. 75 duplicate records were excluded, among which 74 records were excluded by reading the title and abstract, and one record was excluded by reading the full article. 264 articles were excluded (including one registered trial) because they did not meet the inclusion criteria; one article was further excluded because the data of the study cannot be collected from the article and there was no response from the corresponding author. Two studies about acupuncture of LE are ongoing. Finally, four studies [20–23] were included and two ongoing trials [26, 27] were described in this systematic review.

### 3.2. Characteristics of the Included Studies

Characteristics of the methods, participants, intervention, and outcome measures of all included studies and ongoing studies were shown in Tables 1 and 2 separately. Among the four included studies, there were two conducted in China [22, 23], and two were conducted in Germany [20, 21]. In total, there were 309 patients who participated in the included studies, among which there were 155 patients in the acupuncture group and 154 patients in the controlled group. Their age ranged from 18 years to 70 years, and the duration of the LE was varied from one month to 15 months. All studies included both men and women. Two trials [20, 21] compared acupuncture with sham acupuncture, one trial [22] compared electroacupuncture plus moxibustion with material insulation with blockage therapy, and one trial [23] compared electroacupuncture plus blockage therapy with blockage therapy. In these trials, frequency of acupuncture was at least 20 min per treatment and 3 treatments. Four studies [20–23] reported outcomes including the functional status and pain change; three studies [20, 21, 23] reported the myodynamia change as outcome.

### 3.3. Methodological Quality of Included Studies

**Figure 2:**
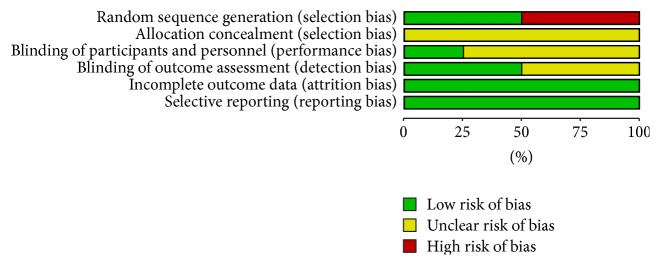
Risk of bias graph: review authors' judgements about each risk of bias item presented as percentages across all included studies.

**Figure 3:**
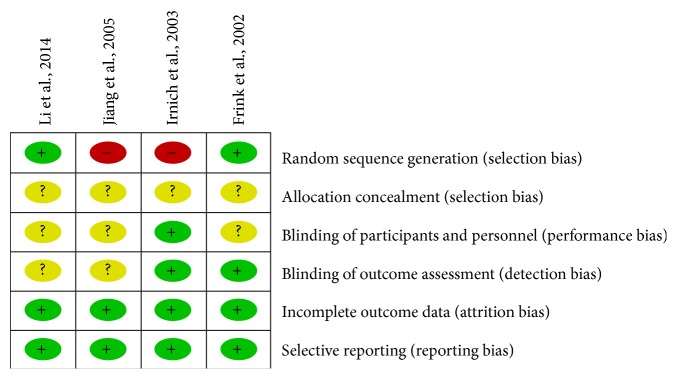
Risk of bias summary: review authors' judgments about each risk of bias item for each included study.

The methodological quality of included studies was assessed by risk of bias, presented in Figures 2 and 3. All of the included studies reported randomization allocation. One study [20] described the method of random sequence generation which was a list random number prepared by the Department of Biostatistics of Hannover Medical School. One study [23] reported the randomization allocation by using Microsoft Office Excel, and another two studies [21, 22] used the randomization allocation method according to sequence of patients' attendance. All of the four studies did not describe the allocation concealment in sufficient detailed ways. Only one study [21] reported the blinding of the participants, and two studies [20, 21] reported the blinding of the outcome assessment, in which one [20] was assessed by an assessor who had no knowledge of acupuncture and another one [21] was assessed by a blinded study nurse. Two trials [21, 22] had low risk of attrition bias, which reported that no participants dropped out or were excluded from the primary analysis, and another two trials [20, 23] also had low risk for reporting the number of dropouts. All of the relevant outcomes were reported in detail in the four trials [20–23], which had low risk of reporting bias. None of the four trials [20–23] reported the source of financial support, declared that no financial interests exist, or mentioned that the research was approved by ethics committee.

### 3.4. Measures of Effect

#### 3.4.1. Elbow Functional Status

**Figure 4:**
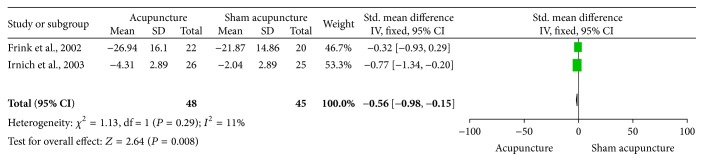
The effect of acupuncture versus sham acupuncture on elbow functional status.

*Acupuncture versus Sham Acupuncture*. The clinical heterogeneity of two trials [20, 21] comparing acupuncture with sham acupuncture is considerable. Pooled analysis showed no statistical heterogeneity among the studies (*P* > 0.1) and was statistically significant (SMD −0.56, 95% CI −0.98 to −0.15, *P* = 0.008), which suggested that the effectiveness of acupuncture on treating function disability of LE is better than sham acupuncture (see Figure 4).

**Figure 5:**
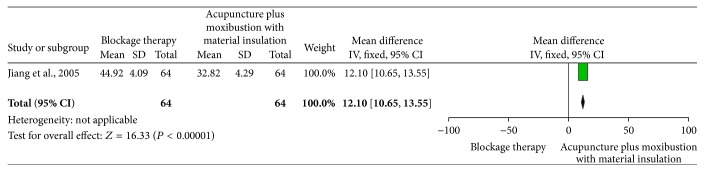
The effect of acupuncture plus moxibustion with material insulation versus blockage therapy on elbow functional status.

*Acupuncture Plus Moxibustion with Material Insulation versus Blockage Therapy*. Only one trial [22] compared the acupuncture plus moxibustion with material insulation with acupuncture alone (MD 12.10, 95% CI 10.65 to 13.55). The result showed that acupuncture plus moxibustion with material insulation is superior for improving the function disability to acupuncture alone (see Figure 5).

**Figure 6:**
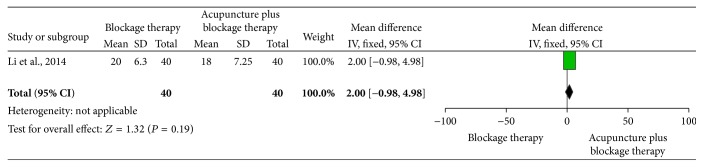
The effect of acupuncture plus blockage therapy versus blockage therapy on elbow functional status.

*Acupuncture Plus Blockage Therapy versus Blockage Therapy*. One trial [23] revealed no statistically significant difference between acupuncture plus blockage therapy and blockage therapy alone (MD 2, 95% CI −0.98 to 4.98), which demonstrated that acupuncture plus blockage therapy was not superior to blockage therapy alone in improving function disability of LE (see Figure 6).

#### 3.4.2. Myodynamia

**Figure 7:**
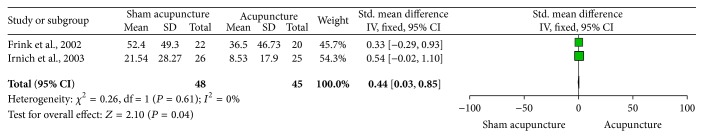
The effect of acupuncture versus sham acupuncture on elbow myodynamia.

*Acupuncture versus Sham Acupuncture*. Two trials [20, 21] were included to pool-analyze the change in myodynamia, which showed that acupuncture group had better effect than sham acupuncture (SMD 0.44, 95% CI 0.03 to 0.85, *P* = 0.04), and the statistical heterogeneity was not significant (*P* = 0.61) (see Figure 7).

**Figure 8:**
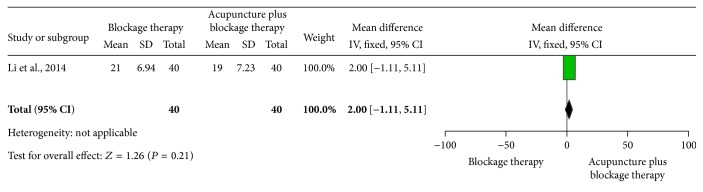
The effect of acupuncture plus blockage therapy versus blockage therapy on elbow myodynamia.

*Acupuncture Plus Blockage Therapy versus Blockage Therapy*. One trial [23] showed no statistically significant difference between acupuncture plus blockage therapy and blockage therapy alone (MD 2, 95% CI −1.11 to 5.11, *P* = 0.21) (see Figure 8).

### 3.5. Adverse Events

Of the four studies, three studies [20, 21, 23] reported the adverse events. One study [20] reported that no serious adverse event was observed during the study. The other two studies [20, 22] reported that the pain would be the main reason for the dropout.

### 3.6. Quality of Evidence

The quality of evidence evaluated using the Grading of Recommendations Assessment, Development, and Evaluation (GRADE) system was from very low to low (Table 3). All of the studies reported the randomization method, but two of the studies [21, 22] used the wrong random method, according to the sequence of attendance. Besides, all of the studies did not describe the method of allocation concealment in a detailed way, and only one study [21] reported the blinding of the participants, which downgraded the outcomes. In addition, the small number of participants of all outcomes also downgraded all outcomes.

## 4. Discussion

Comprehensive search was conducted through 7 electronic databases and WHO International Clinical Trials Registry Platform Search Portal for acupuncture in treating LE. The studies identification, data extraction, and analysis were carried out independently by two review authors. In this systematic review, we included a total of four randomized controlled trials [20–23] with 309 participants suffering from LE. Of all studies, two studies [20, 21] were designed to compare acupuncture with sham acupuncture, one study [22] compared acupuncture combined with moxibustion with material insulation to blockage therapy, and another study [23] was designed to compare acupuncture plus blockage therapy with blockage therapy. In the outcomes, two studies [20, 21] that compared acupuncture with sham acupuncture showed that acupuncture significantly improves elbow functional disabilities and myodynamia. Besides, one trial [22] also showed that electroacupuncture combined with moxibustion with material insulation improved elbow functional disabilities and myodynamia when compared with blockage therapy. However, when combining electroacupuncture with blockage therapy to compare with blockage therapy alone, there is no change in elbow functional status and myodynamia. Up to the present, there were five systematic reviews that have assessed acupuncture for LE. All of the systematic reviews have assessed the effectiveness and safety of acupuncture for LE by evaluating the pain and suggested that acupuncture has some effect on treating elbow pain. Although there are two systematic reviews that have assessed the strength of arm and functional status of arms, they did not draw a definitive conclusion on whether acupuncture is effective for LE or not. Therefore, we conducted this systematic review to assess the effectiveness and safety of acupuncture for LE on elbow functional disability and myodynamia. In the present review, we analyzed four studies accounting for 309 participants on elbow functional status and myodynamia, which showed that acupuncture has some effect on elbow functional disability. While this finding seems promising, it should be interpreted with caution because of the small number of included studies and participants. Besides, there were no trials reporting a formal sample size calculation which is essential to ensure adequate statistical power.

There are still several limitations in this review. The key limitation was the small number of the included studies and participants, which limited the reliability of the pooled results. Another limitation was the quality of the included studies. Mostly included studies were of low quality due to no detailed definition on random sequence generation, allocation concealment, and blinding of participants and personnel. The low quality of the included studies also limited the reliability of conclusion of this review. Besides, all the included studies were from Germany and China, but LE is a worldwide disease. Last, because of ununiformed assessment scale of elbow functional status and upper limb myodynamia, even though SMD method was applied to standardize the results of the studies to a uniform scale before they were combined, we still cannot ignore the report bias caused by the ununiformed assessment scale.

Thus, based on the current systematic review, no firm conclusion can be drawn regarding the effect of acupuncture for LE. Although four randomized controlled trials were identified, the methodological quality of the four studies was low due to high risk in selection bias and blinding of participants and personnel, and the number of the patients recruited in each trial was small, which limited the reliability of the pooled results. Besides, the small number of the included studies also makes the results of this review inconclusive. In the end, in order to confirm the effectiveness of acupuncture for LE, large prospective trials with rigorous design should be conducted more rigorously in the future trials and the assessment scale as well as the evaluation standard needs to be unified from international institute of health as soon as possible.

## Figures and Tables

**Table 1:** The characteristics of included studies in this systematic review.

Author, year	Method	Participants total (*T*/*C*)	Mean (SD)/range age		Intervention		Acupuncture points	Frequency and duration of acupuncture intervention	Outcomes
			Treatment	Control	Treatment	Control			
Fink et al., 2002 [20]	RCT	45 (23/22)	52.5 ± 8.7	51.6 ± 10.0	Real acupuncture	Sham acupuncture	Ashi, LI10, LI11, LU5, LI4, and SJ5	25 min/treatment; 10 treatments, with 2 treatments per week	(1) Maximal muscle strength (2) VAS (3) DASH

Irnich et al., 2003 [21]	RCT	50 (25/25)	31–70	38–65	Real acupuncture	Sham acupuncture	LI4, LI10, SI3, SJ5, and GB34	25 min/treatment; 3 treatments within 10 days	(1) PPT (2) GS (3) IP

Jiang et al., 2005 [22]	RCT	128 (64/64)	42.14 ± 5.62	41.68 ± 5.76	Electroacupuncture plus moxibustion with material insulation	Blockage therapy	LI4, LR3	20 min/treatment; 10 treatments within 5 treatments per week	Elbow function status

Li et al., 2014 [23]	RCT	86 (43/43)	18–22	18–22	Electroacupuncture plus massage and blockage therapy	Blockage therapy	Ashi, LI11, LI12, LI10, SJ5, and LI4	30 min/treatment; once per day for 10 days	(1) VAS (2) GSI (3) MEPS

VAS: Visual Analog Scale; DASH: disabilities of the arm, shoulder, and hand; PPT: pressure pain threshold; GS: grip strength; IP: impairment caused by pain; GSI: grip strength index; MEPS: Mayo Elbow Performance Score.

**Table 2:** The characteristics of ongoing studies in this systematic review.

Title	Country	Trial registration	Inclusion criteria	Exclusion criteria	Method	Participants total	Intervention			Outcomes	
							Treatment		Control	Primary outcome	Second outcome
Acupuncture for Lateral Epicondylitis (Tennis Elbow): Study Protocol for a Randomized Practitioner-Assessor Blinded, Controlled Pilot Clinical Trial	Korea	Clinical Research Information Service (CRIS), Republic of Korea: KCT0000628	(1) Individuals between the ages of 19 and 65 years with lateral epicondylitis on one arm and pain persisting for at least 4 weeks; (2) individuals with tenderness limited to the elbow joint and surrounding area; (3) individuals reporting pain under resisted extension of the middle finger and wrist; (4) individuals with an average pain of 40 or more (0–100) on the Visual Analogue Scale (VAS) in the week prior to the screening visit; (5) individuals who volunteered to participate in the study and who signed a consent form	(1) Individuals whose radiological examinations show abnormalities such as calcification, arthritis, and inflammatory arthropathy of the elbow joint; (2) individuals with a history of trauma, ligament damage, fracture, tumor, or surgery of the elbow joint; (3) individuals who have been diagnosed with or treated for cervical radiculopathy or herniation of intervertebral disc; (4) individuals who have received injections for lateral epicondylitis during the last 6 months; (5) individuals who have received treatments such as nonsteroidal anti-inflammatory drugs (NSAIDs), acupuncture, and physiotherapy for lateral epicondylitis during the last 2 weeks; (6) individuals judged by the person in charge of the clinical trial as unsuitable for participation, such as those with mental disorders, those who are pregnant, or those that have other acute or chronic disorders	RCT	45	Ipsilateral acupuncture group	Contralateral acupuncture group	Control group	The Visual Analog Scale (VAS) at 4 weeks	(1) The Visual Analog Scale (VAS) at 8 and 12 weeks; (2) the patient-rated tennis elbow evaluation (PRTEE); (3) pain-free/maximum grip strength; (4) pressure pain threshold; (5) clinically relevant improvement, patient global assessment, and EuroQol at 4, 8, and 12 weeks

Clinical Comparative Effect of Physiotherapy or Acupuncture Treatment of Lateral Epicondylitis: A Randomized Controlled Pilot Trial	Norway	ClinicalTrials.gov: NCT02321696	(1) Lateral epicondylitis (LE) (duration: >2 weeks); (2) unilateral localization; (3) individuals with average pain of NRS 4 or higher during the last week prior to screening; (4) age between 18 and 67 years; (5) written informed consent	(1) Corticosteroid injections during the last 4 weeks; (2) diseases of the central or peripheral nervous system; (3) inflammatory rheumatic diseases; (4) radioulnar or radiohumeral osteoarthritis; (5) unwillingness to participate	RCT	36	Acupuncture and eccentric exercise	Physiotherapy and eccentric exercise	Watchful waiting and eccentric exercise	Elbow pain on Numeric Rating Scale (0–10)	(1) The disabilities of the arm, shoulder, and hand (quick-DASH); (2) quality of life by EQ-5D; (3) sick listing; (4) patients satisfaction; global perceived effect and satisfaction with treatment; (5) use of analgesics; (6) number of treatment sessions

**Table:** (a) Acupuncture versus sham acupuncture

Quality assessment							Number of patients		Effect		Quality	Importance
Number of studies	Design	Risk of bias	Inconsistency	Indirectness	Imprecision	Other considerations	Acupuncture	Sham acupuncture	Relative (95% CI)	Absolute		
Function (follow-up: 14–60 days, measured with scale, range of scores: 10–20, better indicated by lower values)												
2	Randomized trials	Serious^1^	No serious inconsistency	No serious indirectness	Serious^2^	None	48	45	—	SMD 0.56 lower (0.98 to 0.15 lower)	Low	Critical

Myodynamia (follow-up: 4 to 60 days, measured with scale, range of scores: 10–20, better indicated by higher values)												
2	Randomized trials	Serious^1^	No serious inconsistency	No serious indirectness	Serious^2^	None	48	45	—	SMD 0.44 higher (0.53 to 0.85 higher)	Low	Critical

^1^The trial used the wrong random method, which according to sequence of attendance and the method of allocation concealment is not described.

^2^Total population size is less than 400, and effect size is considered a small effect; the upper or lower confidence limit crosses an effect size of 0.5 in either direction.

**Table:** (b) Acupuncture plus moxibustion with material insulation versus blockage therapy

Quality assessment							Number of patients		Effect		Quality	Importance
Number of studies	Design	Risk of bias	Inconsistency	Indirectness	Imprecision	Other considerations	Acupuncture plus moxibustion with material insulation	Blockage therapy	Relative (95% CI)	Absolute		
Function (measured with scales, range of scores: 10–20, better indicated by higher values)												
1	Randomized trials	Very serious^1^	No serious inconsistency	No serious indirectness	Serious^2^	None	64	64	—	MD 12.10 higher (10.65 to 13.55 higher)	Very low	Critical

^1^The method of allocation concealment is not described.

^2^Total population size is less than 400, and effect size is considered a small effect; the upper or lower confidence limit crosses an effect size of 0.5 in either direction.

**Table:** (c) Acupuncture plus blockage therapy versus blockage therapy

Quality assessment							Number of patients		Effect		Quality	Importance
Number of studies	Design	Risk of bias	Inconsistency	Indirectness	Imprecision	Other considerations	Acupuncture plus blockage therapy	Blockage therapy	Relative (95% CI)	Absolute		
Function (follow-up mean: 12 months, measured with scales, range of scores: 10–20, better indicated by higher values)												
1	Randomized trials	Serious^1^	No serious inconsistency	No serious indirectness	Serious^2^	None	40	40	—	MD 2 higher (0.96 lower to 4.98 higher)	Low	Critical

Myodynamia (follow-up mean: 12 months, measured with scales, range of scores: 10–20, better indicated by higher values)												
1	Randomized trials	Serious^1^	No serious inconsistency	No serious indirectness	Serious^2^	None	40	40	—	MD 2 higher (1.11 lower to 5.11 higher)	Low	Critical

^1^The method of allocation concealment is not described.

^2^Total population size is less than 400, and effect size is considered a small effect; the upper or lower confidence limit crosses an effect size of 0.5 in either direction.

## Appendix

### A. Search Strategies

#### A.1. Search Strategy Used in Cochrane Library

#1 “lateral epicondyle”:ti,ab,kw
#2 lateral epicondylitis:ti,ab,kw
#3 “tennis elbow”:ti,ab,kw
#4 external humeral epicondylitis:ti,ab,kw
#5 Lateral Humeral Epicondylitis:ti,ab,kw
#6 #1 or #2 or #3 or #4 or #5
#7 “acupuncture”:ti,ab,kw
#8 “electroacupuncture”:ti,ab,kw
#9 warm acupuncture:ti,ab,kw
#10 neddle acupuncture:ti,ab,kw
#11 mannual acupuncture:ti,ab,kw
#12 #7 or #8 or #9 or #10 or #11
#13 “controlled clinical trial”:ti,ab,kw
#14 “randomised clinical trial”:ti,ab,kw
#15 “randomised control trial”:ti,ab,kw
#16 #13 or #14 or #15
#17 “animal”:ti,ab,kw and “human”:ti,ab,kw
#18 “animal”:ti,ab,kw
#19 #18 not #17
#20 #16 not #19
#21 #6 and #12 and #20

#### A.2. Search Strategy Used in EMBASE

#1 lateral AND epicondylitis
#2 tennis AND elbow
#3 lateral AND epicondyle
#4 external AND humeral AND epicondylitis
#5 lateral AND humeral AND epicondylitis
#6 #1 OR #2 OR #3 OR #4 OR #5
#7 Acupuncture
#8 electroacupuncture
#9 warm AND acupuncture
#10 neddle AND acupuncture
#11 mannual AND acupuncture
#12 #7 OR #8 OR #9 OR #10 OR #11
#13 controlled AND clinical AND trial
#14 randomised AND clinical AND trial
#15 randomised AND control AND trial
#16 #13 OR #14 OR #15
#17 #6 AND #12 AND #16

#### A.3. Search Strategy Used in PubMed

#1 acupuncture
#2 acupuncture therapy
#3 manual acupuncture
#4 electroacupuncture
#5 acupoint
#6 acupuncture[MeSH Terms]
#7 acupuncture therapy[MeSH Terms]
#8 acupoint[MeSH Terms]
#9 #1 OR #2 OR #3 OR #4 OR #5 OR #6 OR #7 OR #8
#10 Tennis Elbow
#11 Elbow, Tennis
#12 Elbows, Tennis
#13 Tennis Elbows
#14 Epicondylitis, Lateral Humeral
#15 Epicondylitides, Lateral Humeral
#16 Humeral Epicondylitides, Lateral
#17 Humeral Epicondylitis, Lateral
#18 Lateral Humeral Epicondylitides
#19 Lateral Humeral Epicondylitis
#20 lateral epicondylitis
#21 lateral epicondyle
#22 external humeral epicondylitis
#23 epicondylitis
#24 Tennis Elbow[MeSH Terms]
#25 #10 OR #11 OR #12 OR #13 OR #14 OR #15 OR #16 OR #17 OR #18 OR #19 OR #20 OR #21 OR #22 OR #23 OR #24
#26 randomised controlled trial
#27 controlled clinical trial
#28 randomized controlled trial
#29 randomised
#30 randomly
#31 placebo
#32 trial
#33 randomized controlled trial[MeSH Terms]
#34 #26 OR #27 OR #28 OR #29 OR #30 OR #31 OR #32 OR #33
#35 (animals) AND humans
#36 animals
#37 #36 NOT #35
#38 #34 NOT #37
#39 #9 AND #25 AND #38

#### A.4. Search Strategy Used in Sinomed

#1 “*网球肘*”[不*加权*:*扩展*]
#2 *网球肘*
#3 *肱骨外*上*髁炎*
#4 (#3) OR (#2) OR (#1)
#5 “*针刺疗法*”[不*加权*:*扩展*]
#6 “*针刺穴位*”[不*加权*:*扩展*]
#7 “*电针*”[不*加权*:*扩展*]
#8 “*温针疗法*”[不*加权*:*扩展*]
#9 “*针刺*”[不*加权*:*扩展*]
#10 *针刺疗法*
#11 *针刺*
#12 *电针*
#13 *温针*
#14 *针刺穴位*
#15 (#14) OR (#13) OR (#12) OR (#11) OR (#10) OR (#9) OR (#8) OR (#7) OR (#6) OR (#5)
#16 “*随机对照试验*”[不*加权*:*扩展*]
#17 “*随机分配*”[不*加权*:*扩展*]
#18 *随机对照试验*
#19 *随机分配*
#20 (#19) OR (#18) OR (#17) OR (#16)
#21 (#20) AND (#15) AND (#4)
#22 ((#21)) AND (人类[*特征词*])
